# Variation in scent amount but not in composition correlates with pollinator visits within populations of deceptive *Arum maculatum* L. (Araceae)

**DOI:** 10.3389/fpls.2022.1046532

**Published:** 2023-01-09

**Authors:** Eva Gfrerer, Danae Laina, Marc Gibernau, Hans Peter Comes, Anja C. Hörger, Stefan Dötterl

**Affiliations:** ^1^Department of Environment and Biodiversity, Paris Lodron University of Salzburg, Salzburg, Austria; ^2^Laboratory of Sciences for the Environment, Centre National de la Recherche Scientifique (CNRS) – University of Corsica, Ajaccio, France

**Keywords:** *Arum maculatum*, Psychodidae, Sphaeroceridae, floral scent phenotype, intrapopulation variation in floral scent and pollinators, negative frequency-dependent selection

## Abstract

Floral scent is vital for pollinator attraction and varies among and within plant species. However, little is known about how inter-individual variation in floral scent affects the abundance and composition of floral visitor assemblages within populations. Moreover, for deceptive plants it is predicted that intra-population variation in scent can be maintained by negative frequency-dependent selection, but empirical evidence is still lacking. To investigate the ecological and evolutionary relations between inter-individual scent variation (*i.e*., total emission and composition) and floral visitors in deceptive plants, we studied floral scent, visitor assemblages, and fruit set in two populations of fly-pollinated (Psychodidae, Sphaeroceridae; Diptera) and deceptive *Arum maculatum* from Austria (JOS) and northern Italy (DAO). By correlating individual data on floral scent and visitor assemblages, we show that inter-individual variation in floral scent partly explains variation in visitor assemblages. The quantity of floral scent emitted per individual correlated positively with visitor abundance in both populations but explained visitor composition only in DAO, where strongly scented inflorescences attracted more sphaerocerid flies. However, in each population, the composition of floral scent did not correlate with the composition of floral visitors. There was also no evidence of negative frequency-dependent selection on floral scent. Instead, in JOS, more frequent scent phenotypes attracted more pollinators and were more likely to set an infructescence than rarer ones. Our results show that floral scent, despite being key in pollinator attraction in *A. maculatum*, only partly explains variation in pollinator abundance and composition. Overall, this study is the first to shed light on the importance of inter-individual variation in floral scent in explaining floral visitor assemblages at the population level in a deceptive plant species.

## 1 Introduction

The majority of angiosperms are pollinated by animals (Ollerton et al., 2011), which are attracted by a range of floral traits, most prominently by visual (*e.g.*, colour) and olfactory (*i.e.*, scent) cues (Armbruster et al., 2005; Schlumpberger et al., 2009; Glover, 2011; van der Kooi et al., 2019). Contrary to visual cues that attract insect pollinators typically from relatively small distances (Chittka and Raine, 2006), olfactory cues can navigate insects from both small and large distances (Raguso, 2008a; Kaushik et al., 2020). Floral scent bouquets can be simple and composed of only a few compounds (Wiemer et al., 2009), but can also include a complex cocktail consisting of a hundred or even more compounds (Knudsen et al., 2006; Gfrerer et al., 2021).

Floral scent bouquets are known to vary inter-specifically (Levin et al., 2001; Friberg et al., 2013) and, for example, facilitate differential pollinator attraction in co-flowering plant species (*e.g.*, Raguso, 2008b) to promote flower constancy and conspecific pollen transfer (Whitehead and Peakall, 2009; Moreira-Hernández and Muchhala, 2019). Likewise, floral scents vary intra-specifically in both total scent emission and composition, both among (inter-) and within (intra-) populations (Delle-Vedove et al., 2017; Dormont et al., 2019; Friberg et al., 2019). Inter-population variation might result from genetic drift (Delle-Vedove et al., 2017), phenotypic plasticity (Luizzi et al., 2021; de Manincor et al., 2022), and/or regional differences in environmental factors (*e.g.*, climate, nutrient availability) that directly affect the quantity and quality of floral scent emissions (Farré-Armengol et al., 2020). Inter-population variation in floral scent may also be the outcome of local adaptation to abiotic and biotic factors (*e.g.*, climate, pollinators, florivores; Strauss and Whittall, 2006; Caruso et al., 2019; Kuppler et al., 2020; López-Goldar and Agrawal, 2021). Similarly, intra-population (inter-individual) variation in scent can be directly caused by different abiotic (*e.g.*, temperature, nutrient availability, soil condition; Majetic et al., 2009; Friberg et al., 2017) and/or biotic conditions (*e.g.*, florivory, Zangerl and Berenbaum, 2009), but could also be the outcome of evolutionary forces, including selection (*e.g.*, Parachnowitsch et al., 2012; Delle-Vedove et al., 2017; Knauer and Schiestl, 2017; Gfrerer et al., 2021; Opedal et al., 2022). In both rewarding and deceptive systems, intra-population variation in floral scent and other floral traits can positively affect population stability (Waser et al., 1996; Hooper et al., 2005), especially when environmental conditions, such as local pollinator availability, change within seasons and among years (Herrera, 1988).

In general, intra-population variation in floral traits can be maintained, among other factors, by fluctuating selection (Scopece et al., 2017; Sapir et al., 2021), relaxed selection (Juillet and Scopece, 2010; Jacquemyn and Brys, 2020; Zeng et al., 2022) and frequency-dependent selection (Delph and Kelly, 2014; Sapir et al., 2021). Deceptive plant species are expected to show higher levels of such inter-individual variation in attractive floral traits than rewarding species (Salzmann et al., 2007; Ackerman et al., 2011; Sletvold and Ågren, 2014). In deceptive species, high inter-individual variation of traits related to pollinator attraction (*e.g.*, colour, scent) has especially been associated with negative frequency-dependent selection (nFDS) for rarer phenotypes, driven by the pollinators’ learning ability to avoid unrewarding visits (Ferdy et al., 1998; Smithson and Gigord, 2001; Ackerman et al., 2011; see also Ayasse et al., 2000). Such type of selection might be particularly strong in pitfall trap flowers, where a non-rewarding visit does not take just a few seconds but typically several hours to a day (Oelschlägel et al., 2009), with potentially strong negative effects on the fitness of deceived pollinators (Vázquez and Barradas, 2017). By contrast, in rewarding plant species, inter-individual variation in traits involved in pollinator attraction is expected to be lower because of positive frequency-dependent selection for more common phenotypes (Levin, 1972; Smithson and Macnair, 1996; Sapir et al., 2021), driven by the pollinators’ floral constancy (Waser and Price, 1981; Salzmann et al., 2007).

To date, few studies have assessed the evolutionary processes that maintain inter-individual variation in floral scent within populations, and whether this variation affects the identity and frequency of floral visitors and, in consequence, fruit set (Galen et al., 2011; Kuppler et al., 2016; Kantsa et al., 2018). So far, however, there is no empirical evidence of nFDS on scent in deceptive plants (Salzmann et al., 2007; Ackerman et al., 2011; Dormont et al., 2019; Braunschmid and Dötterl, 2020). The only studies that have tested for a relationship between inter-individual variation in floral scent and floral visitors have focused on rewarding plant species. For example, in a common garden experiment with *Sinapis arvensis* (Brassicaceae), no correlation was found between inter-individual variation in the total amount of scent and the number of floral visitors attracted, yet inter-individual variation in floral scent composition allowed predicting floral visitor communities (Kuppler et al., 2016). In *Polemonium viscosum* (Polemoniaceae), high levels of intra-population variation in the amounts of a particular scent compound, 2-phenylethanol, were found, whereas high amounts negatively correlated with both flower damage by ants and bumblebee pollinator visitation, indicating a trade-off between pollinator services and plant defence mediated by 2-phenylethanol emission (Galen et al., 2011). Hence, in rewarding plant species, intra-population variation in floral scent has been demonstrated to have ecological consequences for both antagonistic and mutualistic floral visitors (Galen et al., 2011; Kuppler et al., 2016). With respect to deceptive species, intra-population variation in floral scent has been well-documented (Salzmann et al., 2007; Dormont et al., 2019; Gfrerer et al., 2021), but it is unknown whether and how scent variation affects the frequency and composition of floral visitors.

Here, we use *Arum maculatum* L. (Araceae) as a model species to investigate the relationships between floral scent phenotypes and pollinators, and potential evolutionary processes that maintain inter-individual variation in floral scent within populations. This brood-site deceptive species attracts fly pollinators (Psychodidae, Sphaeroceridae; Diptera) by a strong dung-like floral scent (Lack and Diaz, 1991; Kite et al., 1998; Gfrerer et al., 2021; Laina et al., 2022) and temporarily traps deceived pollinators in a floral chamber, from which they all can be easily collected (Lack and Diaz, 1991; Laina et al., 2022). Main floral scent compounds are, among others, indole, *p*-cresol, 2-heptanone, and *β*-citronellene. Previous work has demonstrated that minor but not the main scent compounds of *A. maculatum* are subject to phenotypic selection (Gfrerer et al., 2021) and that floral scent varies not only among but also within populations in total amount and composition (Diaz and Kite, 2002; Chartier et al., 2013; Gfrerer et al., 2021; Szenteczki et al., 2021). Similar to scent profiles, pollinator abundance and composition also vary among and within populations of this species (Espíndola et al., 2011; Chartier et al., 2013; Szenteczki et al., 2021; Laina et al., 2022), whereby inter-population variation in pollinator composition partly mirrors variation in floral scent (Chartier et al., 2013; Gfrerer et al., 2021). It is not yet clear, however, whether there is a correlation between inter-individual variation in floral scent and floral visitors within populations of *A. maculatum*, and whether scent variation can be explained by nFDS.

In the present study, we take advantage of our previous data on scent (Gfrerer et al., 2021), floral visitors (Laina et al., 2022), and fruit set (Gfrerer et al., 2021; Laina et al., 2022) from two extensively studied *A. maculatum* populations, located in northern-central Austria and northern Italy, respectively. In these previous studies, scent and visitor data were analysed separately. Here, we integrate these data to ask whether intra-population variation in total amount of floral scent and scent composition explains the number and composition of floral visitors attracted to individual plants. Further, we ask whether rarer scent phenotypes within each population attract more pollinators and have a higher fruit set compared to more frequent ones, and thus, whether floral scent is subject to nFDS (see also Violle et al., 2017; Braunschmid and Dötterl, 2020).

As observed in rewarding plant species, we predicted for deceptive *A. maculatum* that at least part of the intra-population variation in insect visitation is due to inter-individual differences in floral scent, and that this variation shapes the interaction between *A. maculatum* and its floral visitors at the population level. In turn, we expected nFDS to explain the high intra-population scent variation observed and, therefore, rarer scent phenotypes having higher fruit set than more common ones.

## 2 Materials and methods

### 2.1 Study plant species and populations

*Arum maculatum* is widespread in most of Europe, with a distributional range extending to northern Turkey and western Caucasus (Boyce, 2006; Govaerts et al., 2020). This rhizomatous perennial herb flowers from April to May, and anthesis lasts less than two days (Lack and Diaz, 1991; Marotz-Clausen et al., 2018). The inflorescence is constructed of a spadix, which consists of an apical appendix (sterile) and the fertile and sterile flowers of both sexes, and is surrounded by a modified bract (spathe) that forms a floral chamber at its basis. In the evening of the first day of anthesis, during the female phase (*i.e*., receptive stigmas), the appendix is highly thermogenic and releases strong scents to attract the pollinators (Bermadinger-Stabentheiner and Stabentheiner, 1995; Gibernau et al., 2004). Once attracted, insects are trapped in the chamber until the second day of anthesis (male phase); loaded with pollen, the insects are released when the spathe withers and are possibly attracted by another female-phase inflorescence (Lack and Diaz, 1991; Gibernau et al., 2004). After successful pollination, the berry-like fruits are retained as an infructescence, with each fruit containing up to five seeds (Sowter, 1949; Lack and Diaz, 1991). In this study, a subset of previously collected data (only flowering season 2017) on floral scent (Gfrerer et al., 2021), visitors (Laina et al., 2022), and fruit set (Gfrerer et al., 2021; Laina et al., 2022) of individual plants were analysed from one population each in northern-central Austria [Salzburg, Josefiau; 47° 46.98’ N, 13° 04.50’ E] and northern Italy [Trentino, Daone; 45° 57.60’ N, 10° 34.80’ E], hereafter referred to as ‘JOS’ and ‘DAO’, respectively. Infructescences were collected in summer 2017 from the same individuals for which scent and insects had been collected in spring of the same year (for details see Gfrerer et al., 2021; Laina et al., 2022). For each infructescence, the number of fruits and unfertilised female flowers was recorded. Fruit set was calculated as the percentage of fruits containing seeds, divided by the total number of female flowers. A single individual usually bears one inflorescence, and only one inflorescence per individual was first sampled in spring and in summer collected as an infructescence.

### 2.2 Insect collection and identification

For the present study, insect data for each population at the individual (*i.e.*, inflorescence) level (*N*_JOS_ = 34, *N*_DAO_ = 61) were obtained from Laina et al. (2022), including only visitors collected in JOS and DAO in April and May 2017. In brief, all insects attracted and trapped in a floral chamber were collected using insect-aspirators on the second day of anthesis, *i.e.*, after the female phase (first day) to avoid interference with pollination, but before insects were released from the inflorescences. As Psychodidae (and particularly females of *Psychoda* spp. *sensu lato*) is the main pollinating dipteran family of this plant species (*e.g*., Lack and Diaz, 1991; Kite et al., 1998; Chartier et al., 2013), we further categorised individuals belonging to this group, as follows. Female Psychodidae were identified to species level based on morphology (Vaillant, 1971; Withers, 1989; Ježek, 1990; Faucheux and Gibernau, 2011), or categorised as ‘unidentified females (Psychodidae)’, when identification was not possible (Laina et al., 2022). Male Psychodidae could not be identified to species level and thus were grouped together [‘males (Psychodidae)’; Laina et al., 2022]. Specimens for which the sex could not be determined were grouped in the category ‘unknown sex (Psychodidae)’. The remaining Diptera, which are probably also pollinators of *A. maculatum* (especially Sphaeroceridae and Chironomidae; Laina et al., 2022), were either identified to family level (Oosterbrock, 2006) or otherwise assigned to the additional category of ‘unidentified Diptera’. Similarly, non-Dipteran individuals (Insecta) were either identified to order level or, when identification was not possible, assigned to the category of ‘unidentified Insecta’ (Laina et al., 2022).

For the statistical analyses (see below), and following Laina et al. (2022), we only used those identified insect groups that had at least three observations across all inflorescences surveyed, *i.e.*, male psychodids, female *Psychoda phalaenoides*, female *P. grisescens*, female *P. trinodulosa*, female *P. zetterstedti*, Ceratopogonidae, Chironomidae, Sphaeroceridae, Sciaridae, Cecidomyiidae, and Coleoptera. Unidentified categories [*i.e.*, unknown sex (Psychodidae), unidentified females (Psychodidae), unidentified Diptera and unidentified Insecta] were included in some but not all statistical analyses (see below).

### 2.3 Plant volatile collection and analyses

Inflorescence scent data collected in 2017 for the JOS and DAO populations (*N*_JOS_ = 34; *N*_DAO_ = 61) were obtained from Gfrerer et al. (2021). In short, scent was collected on the first day of anthesis between 18:00 and 19:30, *i.e*., the time period of peak scent emission (Marotz-Clausen et al., 2018), by bagging each inflorescence in an oven bag (c. 30×12 cm; Toppits, Melitta, Germany) and collecting scent *via* dynamic headspace, following Marotz-Clausen et al. (2018), for five minutes at 200 ml min^-1^ with a vacuum pump (rotary vane pump G12/01 EB, Gardner Denver Austria GmbH, Vienna, Austria). Volatiles were trapped on a mixture of Tenax-TA (mesh 60–80) and Carbotrap B (mesh 20–40; 1.5 mg each; both Supelco, Taufkirchen, Germany), which was filled in quartz glass tubes (length: 2–3 cm; inner diameter: 2 mm). Samples from leaves and ambient air served as negative controls. Obtained scent samples were analysed by thermal desorption–gas chromatography/mass spectrometry (TD-20 coupled with QP2010 Ultra EI GC/MS, Shimadzu Corporation, Kyoto, Japan), and acquired data were analysed in GCMSolution v.4.41 (Gfrerer et al., 2021).

### 2.4 Statistical analyses

#### 2.4.1 Relationships between the total amount of scent and visitor abundance and composition within populations

We constructed linear models (LMs) in R v.4.2.0 (R Core Team, 2021) for each population separately to assess whether the total amount of scent emitted by a plant individual had an effect on absolute abundances of attracted floral visitors. For the linear models, total insect abundances (*log*_10_ +1), including both identified and unidentified insect groups (see above), were employed as the response variable and total scents (*log*_10_) as the explanatory variables. Variables were *log* transformed to reduce skewness in the data sets.

To measure the effect of the total amount of scent emitted per inflorescence on the relative abundance of trapped insects (*i.e.*, floral visitor composition) within populations, we calculated distance-based linear models (DistLM; Legendre and Andersson, 1999; McArdle and Anderson, 2001) in PRIMER v.6.0 (Clarke and Gorley, 2006), again for each population separately. For these analyses, we included only the identified insect groups and used their relative abundances (*i.e.*, total abundance of each insect group divided by the total abundance of all visitors) to calculate a response matrix of Bray–Curtis dissimilarities, while the total amount of scent emitted was used as a predictor variable. For each population separately, non-metric multidimensional scaling (NMDS; based on Bray–Curtis values) was used to display variation in visitor composition among individuals (*i.e*., inflorescences), and to visualise the effect of the total amount of scent on the visitor composition (in case of a significant DistLM outcome). For the above analyses, the sample sizes were *N*_JOS_ = 31 and *N*_DAO_ = 57, as inflorescences with no insects had to be excluded (*i.e.*, calculation of Bray–Curtis values not possible) and one outlier was removed. As a significant effect was detected for DAO (see Results), we retained those insect groups showing the strongest correlations (Spearman’s correlation coefficient > |0.2|) with the first two NMDS axes (*i.e.*, Sphaeroceridae, Sciaridae, Chironomidae, female *P. phalaenoides* and *P. grisescens*, and male psychodids), and further assessed the effect of total scent on the abundance of each insect group separately using linear models (LMs) in R.

#### 2.4.2 Relationship between the relative composition of floral scent and floral visitor composition within populations

We tested for a correlation between the relative composition of floral scent (*i.e.*, absolute amounts of single compounds in relation to total amount of scent per individual; Gfrerer et al., 2021) and the composition of floral visitors (*i.e.*, relative abundance). To this end, we generated Bray–Curtis dissimilarity matrices for each population separately and subjected them to Mantel tests (Pearson correlations, 9,999 permutations), using the R package vegan v.2.5-7 (Oksanen et al., 2019). As for the DistLM analyses, these matrices were calculated using only data of the identified insect groups.

#### 2.4.3 Negative frequency-dependent selection (nFDS) on floral scent

To test for nFDS on scent within each population, we calculated for each individual its scent *rarity*, following Braunschmid and Dötterl (2020). Accordingly, we used the Bray–Curtis dissimilarity matrices based on the relative scent composition and calculated the mean pairwise dissimilarity measures for each individual (*rarity*), with higher values being indicative of a rarer floral scent phenotype (Braunschmid and Dötterl, 2020). We explored the relationship between *rarity* and female reproductive success within each population along two axes. First, we asked whether the *rarity* of a scent phenotype can predict the presence/absence of an infructescence and the fruit set. For individuals for which an infructescence could not be found (11 out of 34 in JOS, and 28 out of 61 in DAO), fruit set was coded as zero. We employed generalised linear models (GLMs) and LMs in R for logistic and linear regressions, respectively, using either *presence/absence of an infructescence* (GLMs) or *fruit set* (LMs) as response variable; in both cases, *rarity* was the explanatory variable. Second, we asked whether rarer scent phenotypes attract more insects within each population. For this reason, we also employed LMs, now with the *sum of all visitors* (*log*_10_ +1) as response variable and *rarity* as predictor variable. As we detected a significant effect between visitor abundance and rarity of relative scent composition in JOS (see Results), we further wanted to know whether this effect might be influenced by the total amount of scent. For this purpose, we tested whether rarity also correlates with the total amount of scent in this population, by performing a LM with *total scent* (*log*_10_) as response variable and *rarity* as predictor variable.

For characterising rarer scent phenotypes, we ran a *randomForest* analysis (Breiman, 2001) on relative scent composition data with *rarity* as the response variable (ntree = 9,999 bootstrap samples, mtry = 16), using the R package randomForest v.4.7-1 (Liaw and Wiener, 2002), both for JOS and DAO. We then extracted the importance measurements (increase in mean squared error of predictions in %) for each volatile organic compound (VOC) to identify those that are most strongly influenced by *rarity*. Plotting relative amounts of these scent compounds against rarity revealed how rare phenotypes were characterised by these single VOCs.

## 3 Results

### 3.1 Intra-population variation of floral visitors

**Figure 1:**
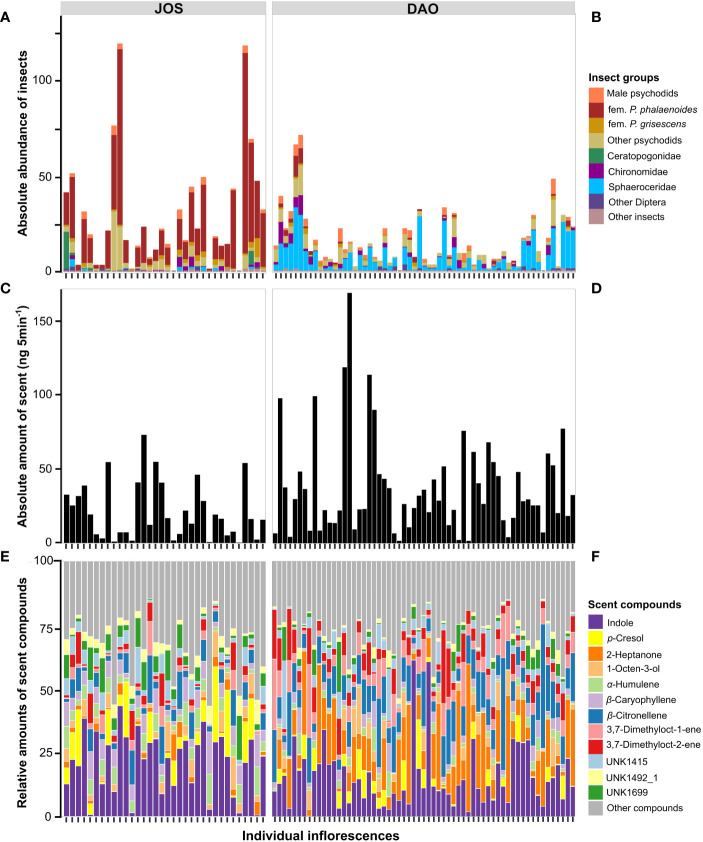
Inter-individual variation in the two studied populations of *Arum maculatum* from northern-central Austria (JOS; Salzburg; *N* = 34; **A, C, E**) and northern Italy (DAO; Daone, Trentino; *N* = 61; **B, D, F**) with respect to **(A, B)** absolute abundance of floral visitors trapped per floral chamber, **(C, D)** total amount of scent emitted, and **(E, F)** relative amounts of scent compounds. fem., female; *P.*, *Psychoda*; UNK, unknown, with the number following UNK being the Kovats' retention index of the compound.

In the two populations of *A. maculatum* studied (JOS and DAO), the number of floral visitors trapped per inflorescence (viz. individual) varied substantially, ranging between zero and 119 insects at JOS (*N*_Total_ = 992 individuals; median = 20 insects) and between zero and 71 insects at DAO (*N*_Total_ = 925; median = 10 insects) (**Figures 1A, B**). In the JOS population, trapped insects consisted mainly of female *Psychoda phalaenoides* (*N*_Total_ = 702 individuals; range = 0–105, median = 13), followed by female *P. grisescens* (*N*_Total_ = 45; range = 0–10, median = 1) and male psychodids (*N*_Total_ = 44; range = 0–5, median = 1) (**Figure 1A**). By contrast, at DAO, the dominating insect groups were Sphaeroceridae (*N*_Total_ = 469 individuals; range = 0–32, median = 5), followed by Chironomidae (*N*_Total_ = 104; range = 0–12, median = 1), male psychodids (*N*_Total_ = 89; range = 0–8, median = 1), female *Psychoda phalaenoides* (*N*_Total_ = 46; range = 0–11, median = 0) and female *P. grisescens* (*N*_Total_ = 25; range = 0–3, median = 0) (**Figure 1B**).

### 3.2 Intra-population variation of inflorescence scent

In the JOS population (*N* = 34), the total amount of scent emitted per individual (*i.e*., ng inflorescence^-1^ 5 min^-1^) varied between 0.6 and 72.7 ng, and in DAO (*N* = 61) between 1.7 and 171.0 ng (**Figures 1C, D**). Individuals in JOS released between 31 and 148 compounds, and in DAO between 23 and 148 compounds. In JOS, the relative scent bouquet was mainly composed of the nitrogen-bearing compound indole (mean = 22.1%; range = 0.6–43.3%), the aromatic *p*-cresol (6.5%; 0–29.5%), the monoterpene *β*-citronellene (6.1%; 0–18.9%), and the sesquiterpenes *β*-caryophyllene (5.9%; 0.1–16.5%) and *α*-humulene (4.8%; 0–11.9%; **Figure 1E**). According to the randomForest analysis, rarer phenotypes were most characterised by higher relative amounts of the irregular terpene 6-methyl-2-heptanone, by lower amounts of indole, the unknown UNK1616, or the sesquiterpene cadalene, or by either very high or no amounts of the monoterpene 3,7-dimethyloct-2-ene, compared to more common phenotypes. In DAO, the scent was mainly composed of indole (15.5%; 0.01–61.2%), the aliphatic compound 2-heptanone (14.0%; 0–57.9%), and the monoterpenes *β*-citronellene (11.6%; 2.5–44.1%), 3,7-dimethyloct-2-ene (6.5%; 0.4–28.8%), and 3,7-dimethyloct-1-ene (6.1%; 0.5–28.6%; **Figure 1F**). Rarer phenotypes were most characterised by higher relative amounts of indole, by lower amounts of the aliphatics 2-heptanol and 3-hepten-2-one, or by either very high or very low amounts of 2-heptanone, compared to more common scent phenotypes.

### 3.3 Effect of total scent emission and scent composition on the (total) abundance and composition of floral visitors attracted

**Figure 2:**
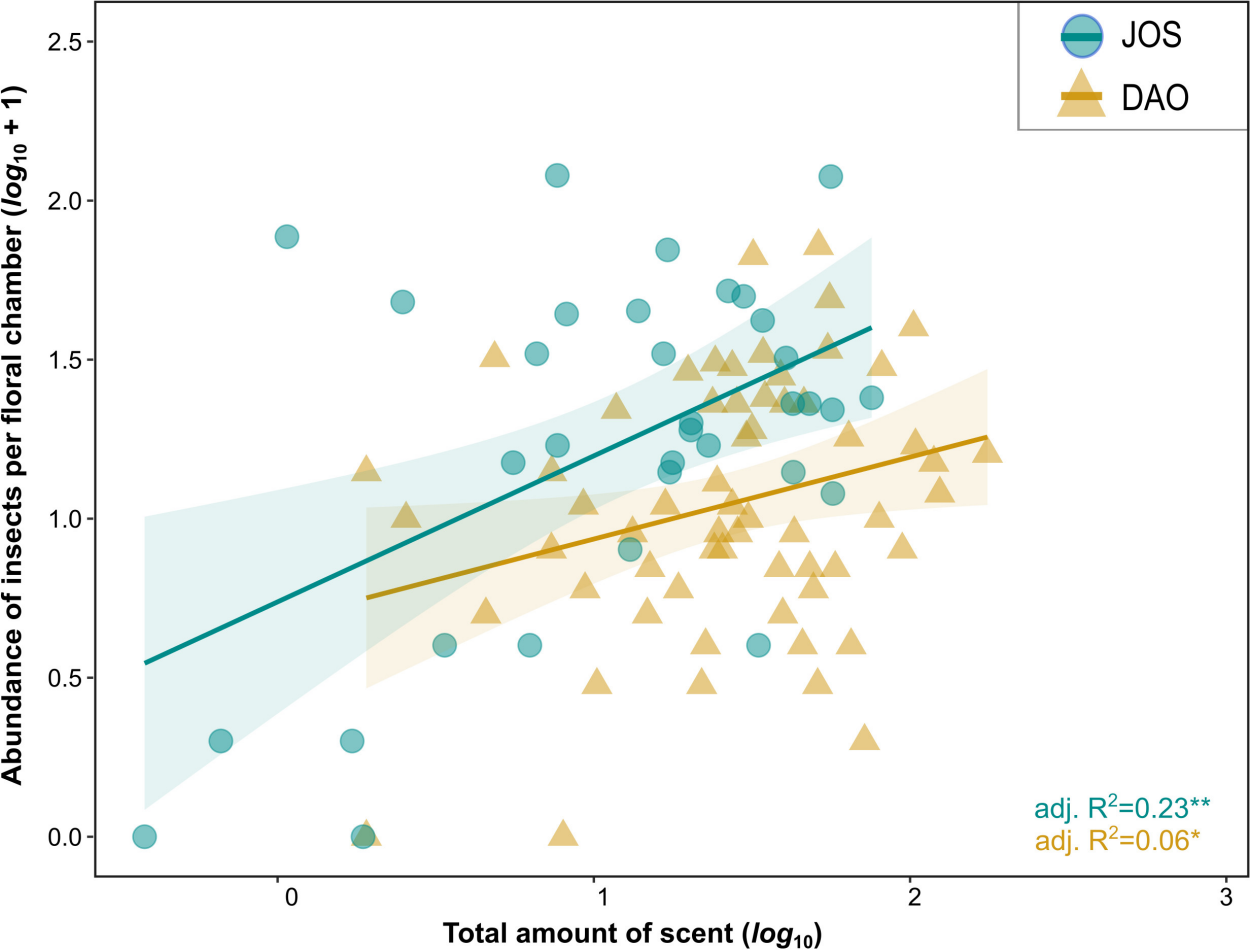
Linear regressions between the absolute abundance of floral visitors (*log*_10_ +1) trapped per floral chamber of *Arum maculatum* individuals and the total absolute amount of floral scent emitted per inflorescence (*log*_10_, ng inflorescence^-1^ 5 min^-1^) in populations JOS (Josefiau, Salzburg, Austria) and DAO (Daone, Trentino, Italy). Solid lines indicate significant model fitting and shaded areas represent 95% confidence intervals. * 0.01 < *P* ≤ 0.05; ** 0.001 < *P* ≤ 0.01.

**Figure 3:**
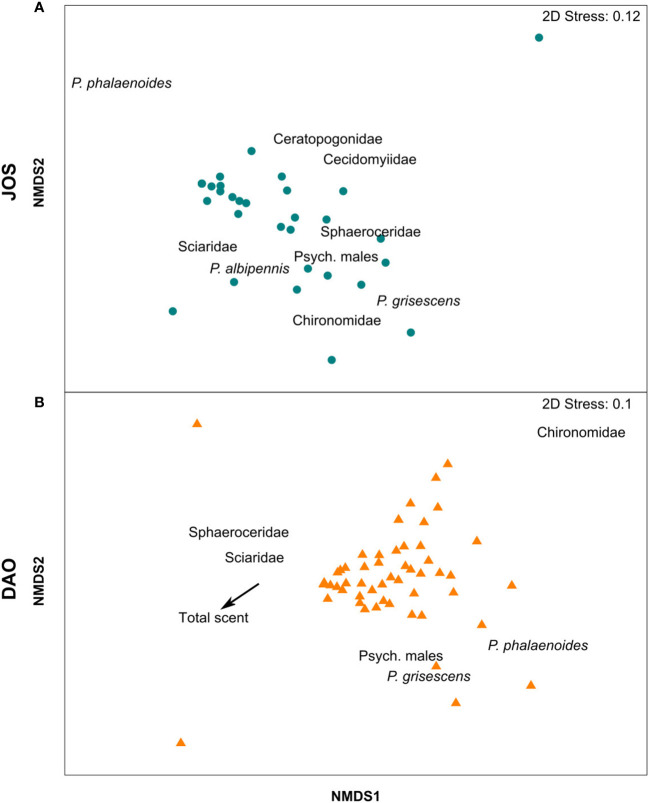
Non-metric multidimensional scaling (NMDS) plots based on Bray–Curtis dissimilarities of relative abundances of insect groups in the two studied populations of *Arum maculatum*: **(A)** JOS (blue dots, *N* = 31) and **(B)** DAO (orange triangles, *N* = 57). Insect groups that correlated most with the axes are displayed (Spearman’s correlation coefficient > |0.2|). In **(B)** the arrow indicates the vector of total scent, which predicted visitor composition in DAO based on DistLM analysis (see text for details). *P.*, *Psychoda*; Psych., Psychodidae.

**Figure 4:**
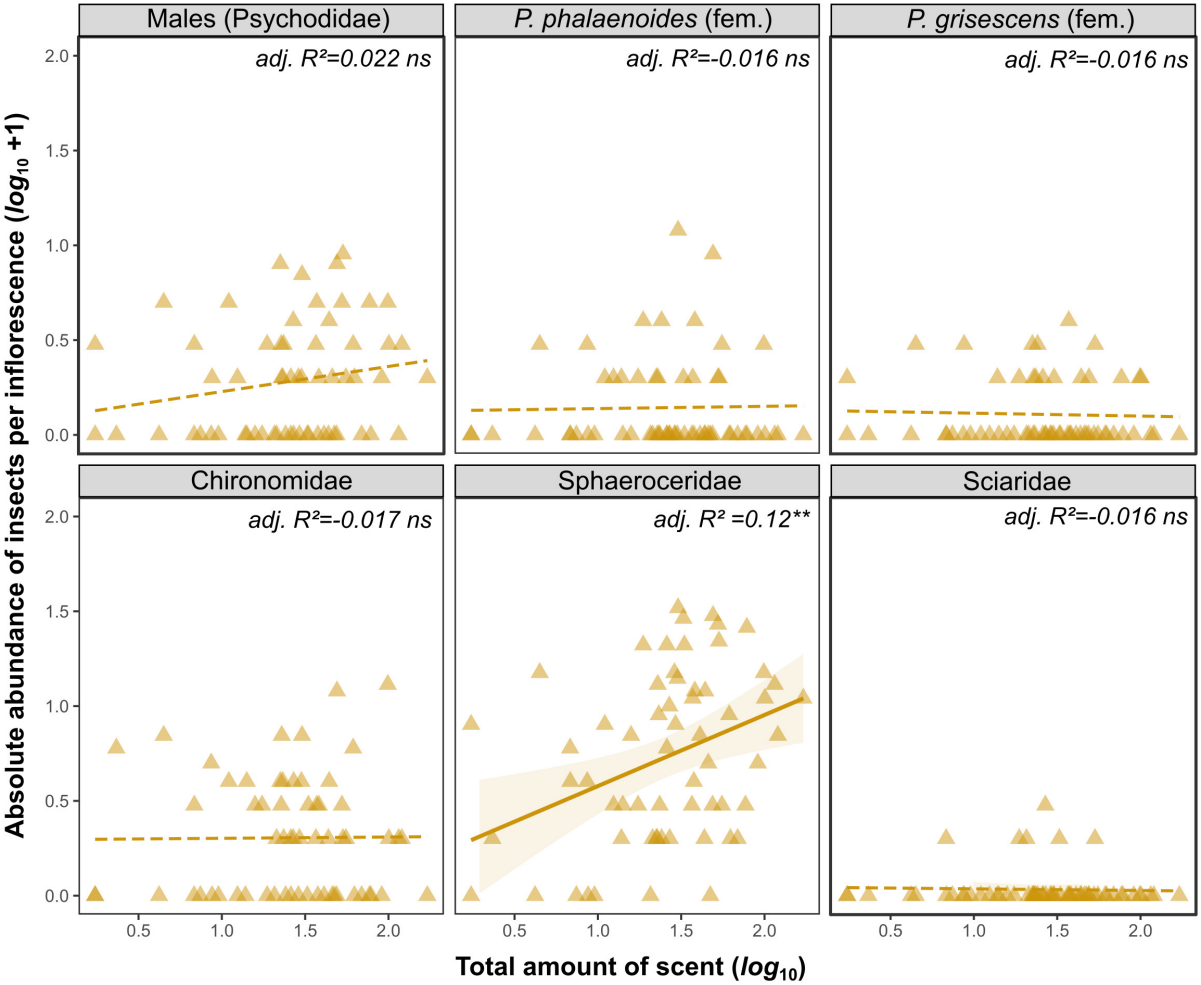
Linear regressions between the absolute abundance of individual insect groups (*log*_10_ +1) and total amount of scent emitted (*log*_10_, ng inflorescence^-1^ 5 min^-1^) per *Arum maculatum* individual for the DAO population (see **Figures 3** and text for details). Solid and dashed lines indicate significant (** 0.001 < *P* ≤ 0.01) and non-significant (*ns*, *P* > 0.05) model fitting, respectively. Shaded areas indicate 95% confidence intervals around regression lines. *P.*, *Psychoda*; fem, female.

In each population, the amount of total scent correlated positively with the number of trapped insects (JOS: slope = 0.45, performance of LM: adj. *R^2^* = 0.23, *df* = 32, *P* = 0.003; DAO: slope = 0.25, adj. *R^2^* = 0.06, *df* = 59, *P* = 0.03; **Figure 2**). The total amount of scent did not predict floral visitor composition in JOS (*Pseudo-F_df=_*_29_ = 0.46, *P* = 0.81; **Figure 3A**), while this was the case in DAO (*Pseudo-F_df=_*_55_ = 3.03, *P* = 0.02; **Figure 3B**). Notably, in this latter population, individuals with higher amounts of scent attracted relatively more sphaerocerids and sciarid flies, while those with lesser amounts of scent attracted relatively more Chironomidae, female *Psychoda phalaenoides*, female *P. grisescens*, and male psychodids (**Figure 3B**). Linear models (LMs) further revealed that the absolute number of Sphaeroceridae attracted by DAO individuals was positively correlated with their total amount of scent (slope = 0.38; adj. *R^2^* = 0.12, *df* = 59, *P* = 0.004; **Figure 4**); however, for each of the other insect groups, the absolute number of individuals did not correlate with total scent amount (adj. *R^2^* ≤ 0.022, all *P* > 0.05). In each population, floral scent composition did not predict the composition of floral visitors, as revealed by Mantel tests (JOS: *Rho* = 0.13, *P* = 0.15; DAO: *Rho* = 0.11, *P* = 0.08).

### 3.4 Rarity as a predictor of floral visitor abundance and reproductive success

**Figure 5:**
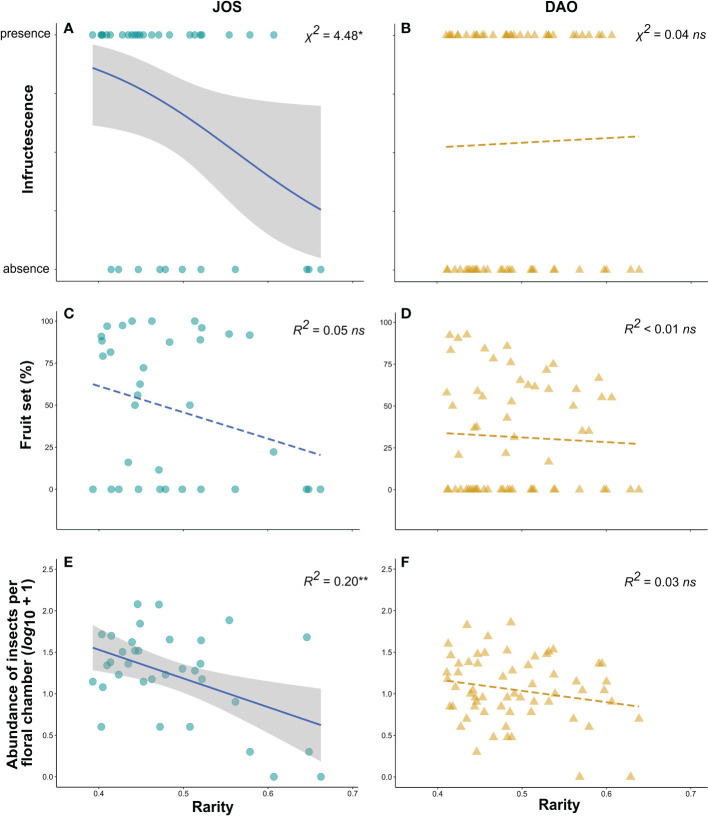
*Rarity* as predictor **(A, B)** of the presence/absence of an infructescence, using logistic regressions, **(C, D)** of fruit set (%), using linear regressions, and **(E, F)** of abundance of insects per floral chamber (*log*_10_ +1), using linear regressions, in the two studied populations of *Arum maculatum* from northern-central Austria (JOS; Salzburg; *N* = 34; **A, C, E**) and northern Italy (DAO; Daone, Trentino; *N* = 61; **B, D, F**). Solid lines indicate significant (* 0.01 < *P* ≤ 0.05; ** 0.001 < *P* ≤ 0.01) model fitting and shaded areas represent 95% confidence intervals. *ns*, non-significant (*P *> 0.05).

In the JOS population, logistic regressions showed that rare scent phenotypes were less likely to set an infructescence (*P* = 0.047, *df* = 32; performance of GLM: *χ*^2^ = 4.48, *P* = 0.034; **Figure 5A**), but no such effect was detected in DAO (*P* = 0.85, *df* = 59; *χ*^2^ = 0.04, *P* = 0.85; **Figure 5B**). Fruit set was highly variable within each population (**Figures 5C, D**), averaging 48% in JOS (*N* = 34) and 31.4% in DAO (*N* = 61) (Gfrerer et al., 2021; Laina et al., 2022). However, in each population, there was no correlation between *rarity* of individual scent phenotypes and fruit set (JOS, performance of LM: adj. *R^2^* = 0.05, *df* = 32, *P* = 0.11; **Figure 5C**; DAO, performance of LM: adj. *R^2^* < 0.01, *df* = 59, *P* = 0.69; **Figure 5D**). *Rarity* was negatively correlated with the abundance of floral visitors in JOS (slope = -3.46; adj. *R^2^* = 0.20, *df* = 32, *P* = 0.004; **Figure 5E**), indicating that rarer scent phenotypes attracted less insects compared to more common ones. Also, in the same population, *rarity* was negatively correlated with the total amount of scent emitted, suggesting that rarer scent phenotypes produce less scent (slope = -6.14; adj. *R*^2^ = 0.58, *df* = 32, *P* < 0.001). A relationship between *rarity* and the abundance of floral visitors could not be detected for DAO (slope = -1.36; adj. *R^2^* = 0.03, *df* = 59, *P* = 0.10; **Figure 5F**).

## 4 Discussion

For each of the two *Arum maculatum* populations studied (JOS, DAO), we found that the total amount of scent predicted the number of attracted floral visitors, *i.e.*, the stronger the floral scent emitted by an individual, the more floral visitors were attracted (**Figure 2**). Further, in the DAO population, but not in JOS, total amount of scent explained floral visitor composition, whereby strongly scented individuals attracted more Sphaeroceridae, in both relative and absolute terms (**Figures 3B**, **4**). In neither population, however, did floral scent composition predict floral visitor composition, nor were rarer scent phenotypes more effective in attracting floral visitors or having a higher fruit set than more common phenotypes (**Figure 5**). Instead, in JOS, but not in DAO, more frequent scent phenotypes attracted more floral visitors and had a higher probability to set an infructescence (*i.e.*, presence/absence) than rarer scent phenotypes (**Figures 5A, E**).

### 4.1 Does variation in floral scent amount and composition explain variation in visitor assemblages?

Our finding that higher total scent amounts result in higher floral visitor abundance (**Figure 2**) differs from the only other study known to us that tested for an effect of inter-individual variation in total scent on the abundance of floral visitors, namely in the rewarding species *Sinapis arvensis*, where no such correlation was detected (Kuppler et al., 2016). Our results, however, are comparable to experimental studies demonstrating that higher amounts of synthetic floral scent compounds attract more insects than lower amounts of such compounds (Theis and Raguso, 2005; Dötterl et al., 2006; El-Sayed et al., 2008). In contrast to weak scent emissions, stronger scents might attract pollinators from greater distances, and also allow them to discriminate more efficiently between (floral) scent cues and olfactory background noise (Beyaert and Hilker, 2014).

In the northern JOS population, where inflorescences mainly attract female *Psychoda phalaenoides* (Laina et al., 2022), the total amount of scent did not affect relative pollinator composition (**Figure 3A**), suggesting that both abundant and less abundant pollinators are efficiently attracted by individual plants that release strong scents. By contrast, in the southern DAO population, total scent amounts predicted not only the total abundance of floral visitors but also their relative composition (**Figure 3B**). Only Sphaeroceridae flies, the most abundant pollinators in this population (Laina et al., 2022), but no other insect group, were more abundant (in absolute numbers) in inflorescences that emitted higher amounts of scent within this population (**Figure 4**). This might suggest that sphaerocerids have a preference for stronger scents and/or are more efficiently attracted from greater distances than other insect groups. Notably, we did not find a positive correlation between the total amount of scent and the abundance of female *Psychoda phalaenoides*, the putative main pollinator of *A. maculatum*, in DAO (**Figure 4**). However here, *P. phalaenoides* is less abundant than in JOS (Laina et al., 2022) and was found as visitor only in about one third of all inflorescences (**Figure 4**), also limiting statistical power. Interestingly, Scheven (1994) reported that, under experimental conditions, *P. phalaenoides* was less attracted to a mixture of synthetic *A. maculatum* scent compounds (*p*-cresol, indole) above a certain concentration threshold. In fact, at high concentrations, indole was not only less attractive to this psychodid species, but it also increased the flies’ mortality rate when experimentally exposed to this compound (Scheven, 1994), as likewise reported for other insect taxa (Cna’ani et al., 2018; Maurya et al., 2020). However, we did not find that strongly scented individuals of *A. maculatum* were less effective in attracting visitors than weakly scented ones (**Figure 2**). It is likely, therefore, that natural scent concentrations of this plant species are lower than those used in experiments, and thus do not negatively affect visitor attraction. Furthermore, in contrast to what has been observed in rewarding *Sinapis arvensis* (Kuppler et al., 2016), we found no effect of inter-individual variation in scent composition on floral visitor composition within the two study populations of deceptive *A. maculatum*. Hence, this variation does not appear to reach the magnitude required to affect the species’ interactions with its fly pollinators.

In general, intra-population variation in floral visitor abundance and composition can be shaped not only by floral scents but also by other phenotypic traits and/or abiotic factors. For example, we know that the inflorescence scent of *A. maculatum* alone efficiently attracts pollinators, while heat alone does not (Kite et al., 1998). However, we cannot exclude that a combination of scent and heat production during anthesis (Bermadinger-Stabentheiner and Stabentheiner, 1995; Marotz-Clausen et al., 2018) influences pollinator behaviour, as for example, by mimicking the temperature of a natural oviposition site (such as fresh dung), resulting in a more efficient luring and trapping of insects (Angioy et al., 2004). In *A. italicum*, also plant size had a positive effect on reproductive success and on the number of trapped insects (Méndez and Obeso, 1993; Albre and Gibernau, 2008). In *A. maculatum*, however, no correlation between inflorescence size and the number of attracted insects was found (Chartier and Gibernau, 2009). In line with this finding, there is no evidence that inflorescence size correlates with scent emission and abundance of floral visitors in the JOS population (no data available for DAO; Gfrerer & Laina, unpublished data). In addition, fluctuations in insect availability and weather conditions during flowering can affect floral visitation (Herrera, 1989; Wilcock and Neiland, 2002). Yet, for our study populations, both explanations seem quite unlikely, as sampling was performed during a short period of peak flowering (seven days in JOS, four days in DAO) under relatively stable weather conditions (*i.e*., minor temperature fluctuations and lack of rain). Instead, for a given plant population, the height of the surrounding vegetation (Sletvold et al., 2013), microhabitat conditions and/or the availability of insect breeding substrates could play a key role in shaping inter-individual variation in the abundance and composition of floral visitors (Roháček et al., 1990; Herrera, 1995; Rodríguez-Rodríguez et al., 2017; Ohler et al., 2020; Arroyo-Correa et al., 2021). These latter factors could be particularly relevant if insects have only limited flight capacities, such as psychodids (Ruthledge and Gupta, 2002; Pech-May et al., 2018), but nothing is known about sphaerocerids in this regard.

### 4.2 Is floral scent in *Arum maculatum* under negative frequency-dependent selection?

Contrary to our prediction, negative frequency-dependent selection (nFDS) was not detected in either of the two populations (**Figures 5C, D**), which is consistent with what has been observed in other deceptive plant species (Salzmann et al., 2007; Ackerman et al., 2011; Braunschmid and Dötterl, 2020). Instead, in one of the populations (JOS), we found a potential signature of positive frequency-dependent selection, in that individuals with more common scent phenotypes were visited by more insects (**Figure 5E**) and had a greater probability of setting an infructescence (presence/absence; **Figure 5A**). Usually, positive frequency-dependent selection is expected to occur in rewarding plant species, where it results in low trait variation and promotes flower constancy of pollinators (Ackerman et al., 2011; Sapir et al., 2021). In JOS, by far the most frequently observed pollinators were females of *P. phalaenoides* (Laina et al., 2022), which preferentially visited common scent phenotypes (**Figure 5E**). In addition, in the present study, we observed that more common scent phenotypes are associated with a stronger scent emission. It is therefore conceivable that these scent phenotypes fit the flies’ olfactory preferences better than rarer phenotypes and/or they were more attractive to the flies because they were stronger scented. If the high visitation rate of common scent phenotypes is (at least in part) due to multiple floral visits of different individuals by individual flies, our findings would also suggest that pollinating flies do not learn to avoid visiting these scent phenotypes. Indeed, these short-lived fly pollinators might have very limited learning capacities (Renner, 2006). Hence, we conclude that nFDS cannot explain the high scent variation observed in the JOS population (**Figures 1E**, **5C**), and other evolutionary processes might be invoked to maintain this variation (*e.g.*, relaxed selection; Juillet and Scopece, 2010; Jacquemyn and Brys, 2020). Likewise, also in the DAO population, inter-individual variation in scent cannot be explained by nFDS. Individuals of this population were observed to have lower fruit set than those in JOS (Gfrerer et al., 2021) and attracted a more variable fly community (in terms of species composition and abundance; Laina et al., 2022). Olfactory preferences are likely to differ between these fly species (Gfrerer et al., 2022), and their availability probably varies from year to year (as demonstrated for other populations of *A. maculatum*; Szenteczki et al., 2021). Overall, this raises the possibility that variation of relative scent composition in DAO (**Figure 1F**) could be subject to different selection pressures by fly pollinators (see also Chartier et al., 2013; Szenteczki et al., 2021; Gfrerer et al., 2022). However, we caution that other than pollinator-related factors can maintain variation in floral scent and affect the reproductive success of plant species, such as resource limitation (Albre et al., 2003), herbivory (Knauer and Schiestl, 2017; Ramos and Schiestl, 2019; Rusman et al., 2019), and potential seasonal variation in floral scent (Dorey and Schiestl, 2022).

## 5 Conclusions

We demonstrated that inter-individual scent variation within populations of *A. maculatum* only partly shapes the interaction with floral visitors, even though scent is the key pollinator attractant of this plant species (Kite et al., 1998). Also, we found no evidence that inter-individual scent variation in *A. maculatum* is maintained by nFDS. Instead, in one of the two study populations (JOS), more frequent scent phenotypes attracted more visitors and were more likely to set an infructescence than rare scent phenotypes, although fruit set was independent of scent *rarity*. Future studies should seek to elucidate the molecular mechanisms underlying the observed scent variation in *A. maculatum* as well as the learning capabilities of its fly pollinators, potentially negative fitness effects of deception on the flies. In general, studies of additional plant species that release variable scents are needed to better understand the relative importance of different evolutionary procecces and ecological factors (Ramos and Schiestl, 2020) that maintain and/or generate intra-population scent variation. Also it would be interesting to test whether pollinator-generalist plant species, which target several pollinators with likely different olfactory preferences, are generally more variable in scent than pollinator-specialists. Finally, Gfrerer et al. (2022) used a subset of five floral visitor species to determine physiologically active scent compounds of *A. maculatum*. When having such data available for not only some but for most of the visitor species, it would be feasible to test whether physiologically active versus inactive compounds i) differ in levels of intra-population variability, ii) have differential explanatory power in predicting flower visitor abundance and composition (high versus low), and iii) are shaped by different evolutionary forces (selection versus drift).

## Data availability statement

The original contributions presented in the study are included in the article. The full scent dataset can be found in the Dryad Digital Repository (https://doi.org/10.5061/dryad.pnvx0k6kn). Further inquiries can be directed to DL, danai.laina2@plus.ac.at or SD, stefan.doetterl@plus.ac.at.

## Author contributions

SD, MG, ACH, and HPC conceived the study. All authors designed the study. EG and DL collected the data, performed all analyses, and wrote the first draft of the manuscript. All authors contributed to the current manuscript version.
